# Patient and nurse preference for Sensoready autoinjector pen versus other autoinjectors in multiple sclerosis: results from a pilot multicenter survey

**DOI:** 10.1186/s12883-023-03100-1

**Published:** 2023-02-27

**Authors:** Amy Perrin Ross, Christian Besser, Shubhanvita Naval, Dee Stoneman, Harriet Gaunt, Noreen Barker

**Affiliations:** 1 Neuroscience Nurse Consultant, Oak Brook, IL USA; 2 Novartis Pharma AG, Basel, Switzerland; 3 Novartis Healthcare Pvt. Ltd, Hyderabad, India; 4 Adelphi Research, Bollington, UK; 5 University College London Hospitals, London, UK

**Keywords:** Autoinjectors, Sensoready pen, Patient preference, Nurse preference, Ofatumumab

## Abstract

**Background:**

Sensoready® autoinjector pen facilitates self-administration of subcutaneous ofatumumab injections at home. We aim to investigate patient and nurse preference for using Sensoready® versus comparator autoinjectors in multiple sclerosis (MS).

**Methods:**

A pilot survey was conducted in Germany followed by in-field interviews across United States, Germany, France, and Italy. The survey recruited 80 MS patients and 50 MS nurses. Respondents were interviewed for 45-min on qualitative open-ended and quantitative close-ended survey consisting of 31 questions for patients and 41 for nurses. Ratings were measured on Likert scale from 1 (not at all important) to 10 (extremely important).

**Results:**

“Easy to perform self-injection with the pen” and “Patient able to use independently” (both, mean overall score 9.4) were the most important attributes for both patients and nurses. Sensoready® scored high across most important attributes for both patients and nurses (*p* < 0.05). Sensoready® was preferred over comparator devices across majority of the important attributes (84%; *p* < 0.05), especially ease of use of the pen (mean overall score 9.4). Sensoready® was preferred over their current device by 9/10 nurses and 8/10 patients if they had to choose a treatment based on the device alone.

**Conclusion:**

Both MS patients and nurses preferred the Sensoready® (ofatumumab) over comparator autoinjectors for their treatment, mostly driven by ease of administration.

## Introduction

Multiple sclerosis (MS) is a complex neurodegenerative disorder of the central nervous system, which typically affects young adults [1]. B cells have essential functions in regulating the immune response and may contribute to the pathogenesis of MS by regulating the T-cell activation process via antigen presentation, cytokine production, oligodendrocyte and neuronal injury contributing soluble toxic factors production, formation of ectopic germinal centers and providing a reservoir for Epstein–Bar virus infection in addition to producing antibodies [2]. Ofatumumab, a fully human anti-CD20 monoclonal antibody [3–5], depletes CD20 + B cells in the blood and lymphoid tissues through complement-dependent cytotoxicity and antibody-dependent cell-mediated cytotoxicity [6, 7].

Ofatumumab has been approved in the United States (US) [8], Europe [9], and several other countries for the treatment of relapsing forms of MS in adults based on the results of Phase 3 pivotal ASCLEPIOS I and II trials. In these trials, ofatumumab 20 mg administered subcutaneously in a monthly dosing regimen demonstrated superior efficacy compared with teriflunomide 14 mg orally once daily with a favorable safety profile in patients with relapsing MS [6].

The phase 2 APLIOS study demonstrated the pharmacokinetic bioequivalence of ofatumumab 20 mg administered subcutaneously in the abdomen using an autoinjector (i.e., Sensoready® pen) versus a prefilled syringe [10]. Results showed that using a prefilled syringe assembled with an autoinjector (Sensoready® pen) facilitated self-administration of ofatumumab 20 mg subcutaneously at home, and thus, may help reduce the treatment burden [10]. Besides efficacy and safety, convenience and safe delivery of self-administration via autoinjectors are the key considerations for the treatment of patients with MS. The convenience of treatment administration plays an important role in patient satisfaction and, consequently, adherence [11]. Long-term adherence to injectable MS therapies is generally suboptimal because of various reasons and ultimately impacts the efficacy of these treatments, healthcare resource utilization and healthcare costs [12–15]. Compared with manual injection, auto injection devices available for different disease-modifying therapies (DMTs) improve injection tolerability and patient satisfaction [12]. Injectable DMTs for MS are administered via different types of injection mechanisms such as prefilled syringes, mechanical autoinjectors, and electronic autoinjectors; each type has benefits/features of autoinjectors that may impact patient satisfaction and treatment adherence [16]. A number of studies have shown patient preference for autoinjectors over prefilled syringes or vials with syringes; autoinjectors were more convenient, easier to use, less painful, and required less time to administer [17–19]. To date, there is a lack of data directly comparing the various autoinjectors available for MS. This multicenter survey investigated patient and nurse preference for using the Sensoready® autoinjector pen for subcutaneous administration of ofatumumab 20 mg versus autoinjectors used for other DMTs in MS.

## Materials and methods

### Design of pilot survey

The survey comprised of three core phases:

- **1. Pre-testing phase (involving MS Nurses as research partners):**

Nurse representatives were involved in the review of the survey design, which included providing feedback on the survey materials. As part of the pre-testing phase, preparatory work was conducted in collaboration with MS Nurses (e.g., Nurse representatives with the conditions of interest, representing the countries involved in the survey) who had reviewed and advised Novartis on the design of the survey, list of attributes that were collected, consent language, and instructions.

### Pre-test pilot interviews in Germany

A small-scale pilot survey prior to the roll-out of the wider fieldwork was conducted with patients and nurses who meet the eligibility criteria, to validate and refine the survey materials. The pilot pre-test comprised of a 45-min interview, and a 15-min telephone debrief to ensure full comprehension of the interview content including stimuli, terminology, attributes, and instructions.

- **2. Main survey phase:**

Data collection were via a multi-country (US, Germany, France, Italy) face-to-face 45-min semi-structured in-field interviews. Primary data on patient preference was collected as well as bespoke questions outside of the preference questions.

- **3. Data analysis phase:**

Analysis and interpretation of the results, and external communication.

### Patient population

Patients with relapsing–remitting MS aged > 18 to < 60 years (diagnosed < 15 years ago) who had self-administered their DMT through a subcutaneous/intramuscular injection via an autoinjector for more than 2 months were included. The patients were receiving one of the following 6 injectable treatments—Rebidose/Rebismart (Rebif), Avonex pen (Avonex), Autoject/YpsoMate (Copaxone), and Plegridy pen (Plegridy)—and brought their device to the interview.

Specialist MS nurses, general neurological nurses, or nurse practitioners (US only) who had an experience of 3–35 years in their current role in different practice settings, spent ≥ 80% time in direct patient care, and were experienced in training patients on ≥ 2–6 MS autoinjector devices (Rebidose/Rebismart [Rebif], Avonex pen [Avonex], Autoject/YpsoMate [Copaxone], Plegridy pen [Plegridy]) were recruited. Nurses compared Sensoready® autoinjector pen with any 2 of these mentioned devices.

The survey directly compared the Sensoready® autoinjector pen with 6 injectable DMTs (devices), namely, Rebidose/Rebismart (Rebif), Avonex pen (Avonex), Autoject/YpsoMate (Copaxone), and Plegridy pen (Plegridy), as these devices are widely used across the US, Germany, France, and Italy.

The attribute list included in the survey research was predefined and adapted from the previous studies on ExtaviPro [20] and a survey on patients and nurse preferences for rheumatoid arthritis [21]. Adaptations took place through the advice of MS Nurse Research Partners and adapted following the pilot interviews. They were identified as key attributes to measure across all MS devices and were validated by an MS nurse.

### Ethics approval, consent to participate and consent for publication

The survey was conducted in accordance with the market research guidelines as per European Pharmaceutical Marketing Research Association (EphMRA). According to the latest EphMRA guidelines, any market research does not require Clinical Research Ethics Committee or Independent Review Board approval. Therefore, no formal ethics committee and IRB approval was required [22]. All survey results were anonymized for the purpose of publication. In this report, the use of “patient” or “patients” refers to the feedback provided by the patient participants without attribution to any named individual. Participation was voluntary, and participants were entitled to withdraw at any stage of the process, or subsequently to ask that part or all of the record of their interview was destroyed or deleted. Adequate data protection was ensured, with data access strictly limited to the participants, Novartis, and the survey support team. All participants provided written informed consent to participate in the survey.

### Survey questionnaire

For the main survey, respondents were interviewed for 45 min on quantitative close-ended preference questions (31 for patients; 41 for nurses) and bespoke qualitative open-ended questions. The quantitative ratings were measured on a Likert scale from 1 (not at all important) to 10 (extremely important), allowing respondents to choose their preferred attributes and devices, and were designed to reflect real-world decisions and tradeoffs. Bespoke qualitative questions were in place to assess socio-demographics, clinical characteristics (including disease severity) and current treatment satisfaction.

All respondents were provided with instruction leaflet and given administration instructions in local language to use the Sensoready autoinjector pen as a dummy device.

The present analysis categorized the survey responses into the following sections: (1) Important predefined attributes, where respondents were asked to rate the most important aspects of an injectable device; (2) Comparison of the Sensoready® autoinjector pen with other autoinjectors per pre-defined attributes, where respondents were asked to score and provide a preference rating versus comparator autoinjector devices; (3) Overall device preference, where respondents were asked to rate their overall preference for and satisfaction with the Sensoready® autoinjector pen versus other autoinjectors.

### Statistical analysis

A paired *t*-test was used to test for significant differences between the importance/performance scores. An independent sample *t*-test was performed to determine whether there were significant differences between the scores given by patients and nurses [23]. The chi-square and exact tests were used to determine whether the proportions of respondents answering “Sensoready® autoinjector pen (ofatumumab) performs better than existing devices” and “proportion of respondents who chose Sensoready® autoinjector pen as their preferred device” was significant. All the tests were conducted using the SPSS software.

## RESULTS

### Survey participants

The survey recruited 80 MS patients (average age, 43 years [range: 18–59] and disease duration, 7 years ﻿[1–14] and 50 MS nurses (average practice, 15 years [4–34] and average relapsing–remitting MS patient workload per month, 38 patients [5–200]) at office-based and/or hospital-based practices (82%) and specialist MS centers (18%). The patients had spent an average of 4.6 years (range: 2 months–14 years) on their current device and had received an average of 2 treatments (range: 1–4) previously. Patients had an average of at least 2.7 years of experience using DMTs via an autoinjector across countries. On average, it took patients less than 5 min to read the instruction leaflet and administer a dummy injection using the Sensoready® pen device.

### Important predefined device attributes

**Fig. 1:**
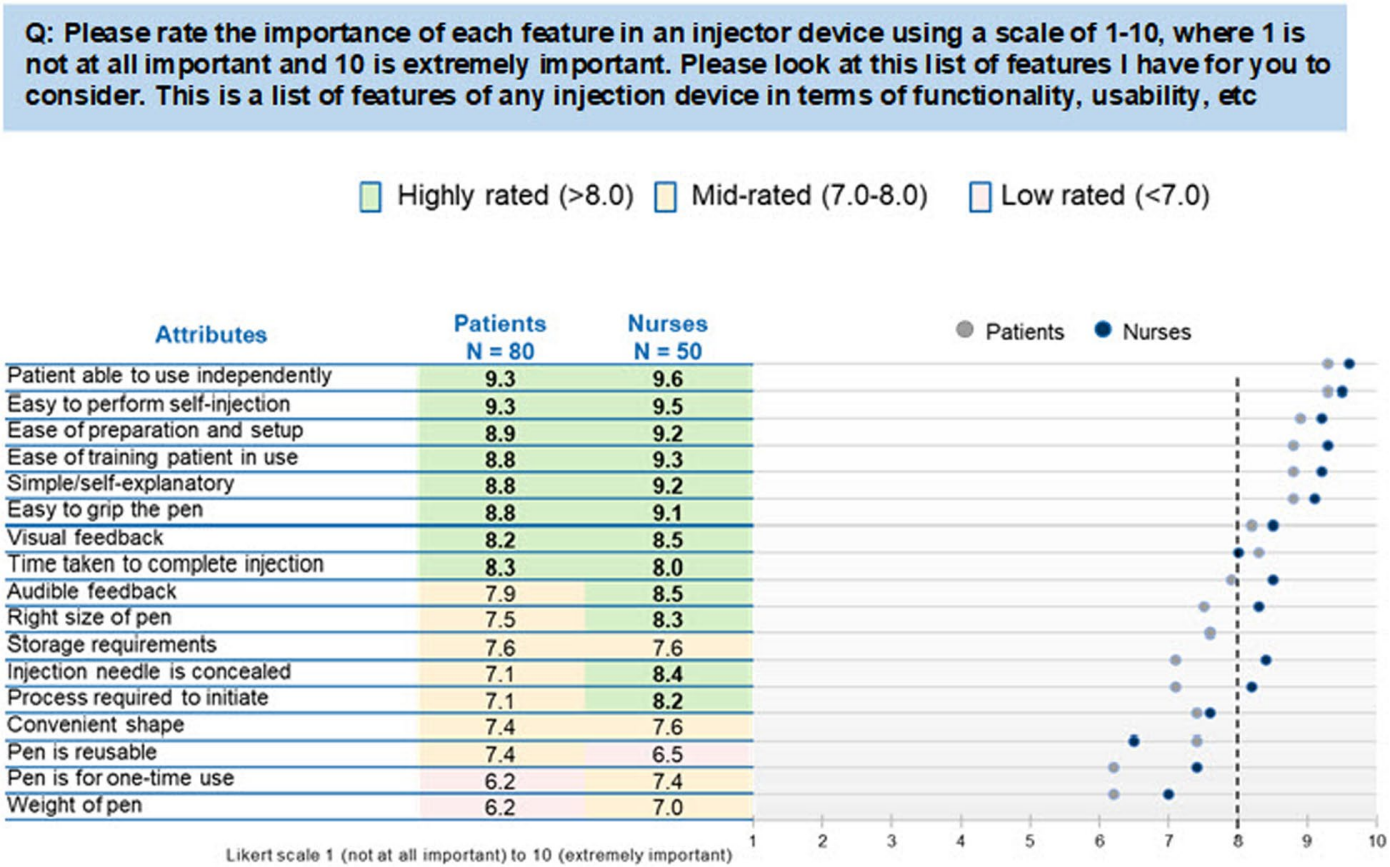
Patient and nurse ratings of importance of attributes of an autoinjector. AI, autoinjector

Patient and nurse ratings of importance of attributes for an autoinjector are presented in Fig. 1. The 2 top highly ranked device attributes for autoinjectors by both patients and nurses were “easy to perform the self-injection with the pen” and “patient able to use independently.” These attributes were ranked significantly higher (*p* ≤ 0.05) than the other attributes and were awarded the highest mean overall score (9.4; maximum possible score, 10.0). The mid-ranking attributes also judged of some importance were “ease of preparation and set-up,” “ease of training patient in use,” “simple/self-explanatory,” and “easy to grip the pen.” Reusability and weight of the device were considered of least importance, particularly for patients.

The mean scores for the attributes by both patients and nurses indicated that the range of the scores was narrower for the highest-ranked attributes (e.g., easy to perform self-injection: 95% top 3 box [scored ≥ 8/10] and 1% bottom 3 box [scored ≤ 3/10]) than for the lowest-ranked ones (e.g., pen is reusable 52% top 3 box, 15% bottom 3 box) [24, 25].

Compared with the overall mean scores, there was a tendency for higher mean importance scores to be awarded by nurses. The mean and median scores throughout the analysis were similar.

Attributes pertaining to patient independence, such as “easy to perform self-injection with the pen” and “patient able to use device independently,” were important for both patients and nurses.

The following attributes were scored as significantly more important by nurses versus patients (all *p* < 0.05): “injection needle is concealed” (mean score 8.4 vs. 7.1), “right size of the pen” (mean score 8.3 vs. 7.5), “process required to initiate” (mean score 8.2 vs. 7.1), “pen is for one-time use” (mean score 7.4 vs. 6.2), and “ease of training patients in use” (mean score 9.3 vs. 8.8).

### Comparison of Sensoready® autoinjector with other autoinjectors

**Fig. 2:**
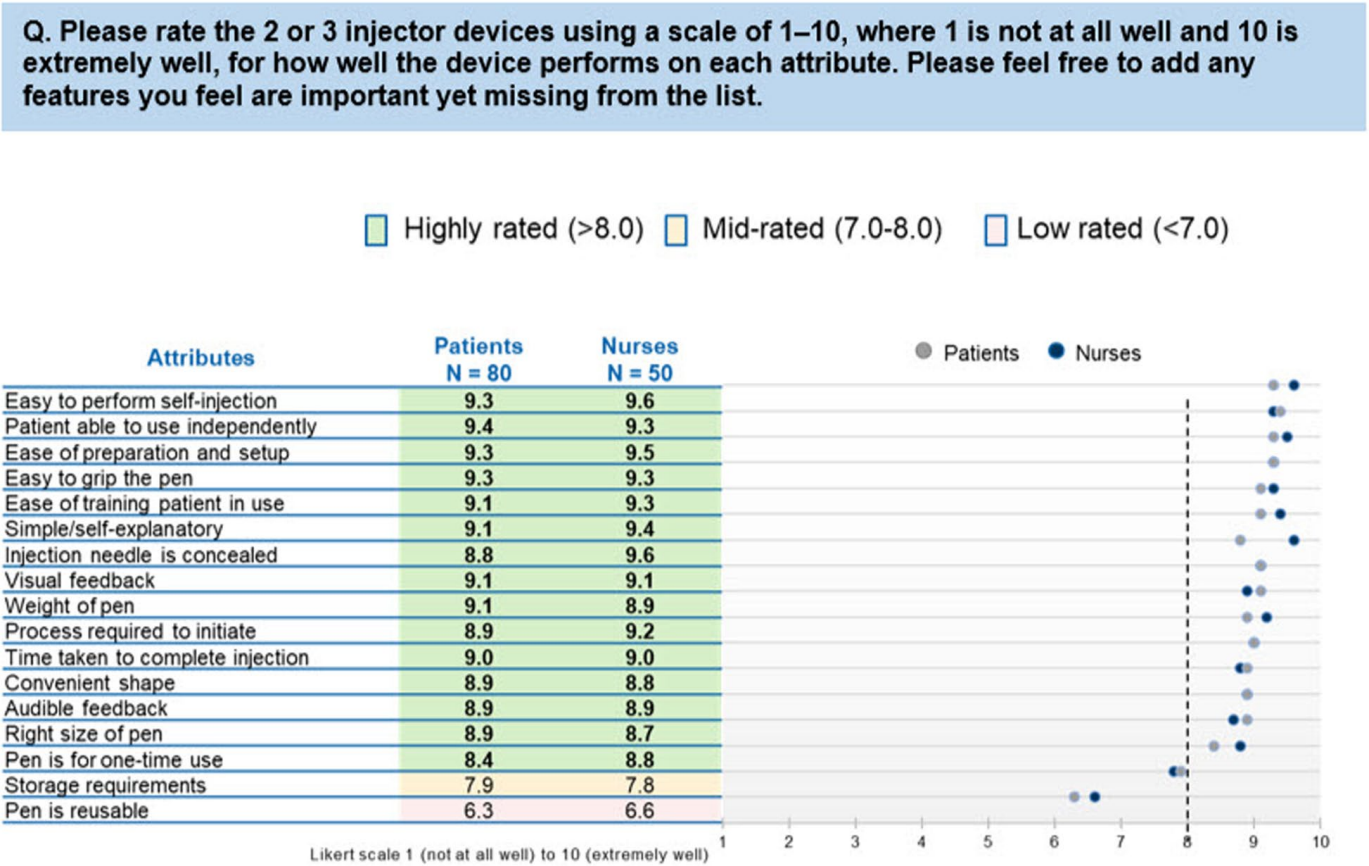
Comparison of Sensoready® autoinjector with other autoinjectors across attributes as rated by patients and nurses. AI, autoinjector. Note: Other devices included were Rebif (Rebidose), Avonex (Avonex Pen), Copaxone (Autoject), Copaxone (YpsoMate), Rebif (RebiSmart), and Plegridy (Plegridy Pen). Of note, Sensoready® AI pen was not used to inject/self-administer medication during the survey nor did all the nurses and patients compare the Sensoready® AI pen against each of the other AI devices

Sensoready® pen (ofatumumab) achieved higher mean scores across majority of the attributes (> 8.0 out of a possible 10) than the comparator autoinjectors and was given similar scores by both nurses and patients. The highest-ranked attributes for Sensoready® pen were “easy to perform the self-injection with the pen” (mean score 9.4), “patient able to use independently” (mean score 9.4), and “ease of preparation and set-up” (mean score 9.4), which were significantly higher than the “mid” and “low” ranking attributes for ofatumumab (*p  *< 0.05). The score for the lowest ranking attribute “pen is reusable” (mean score 6.4) was significantly lower (*p* < 0.05) than that for all other attributes for ofatumumab (Fig. 2). Considering the mean scores by attribute from both patients and nurses, the range was narrower among the highest-ranked attributes than among the lowest-ranked ones. The ofatumumab device achieved similar scores across attributes for both patients and nurses. The biggest variance in mean scores appeared for “injection needle is concealed,” where the Sensoready® autoinjector pen was scored significantly higher by nurses (mean score 9.6) than by patients (mean score 8.8; *p* < 0.05).

**Fig. 3:**
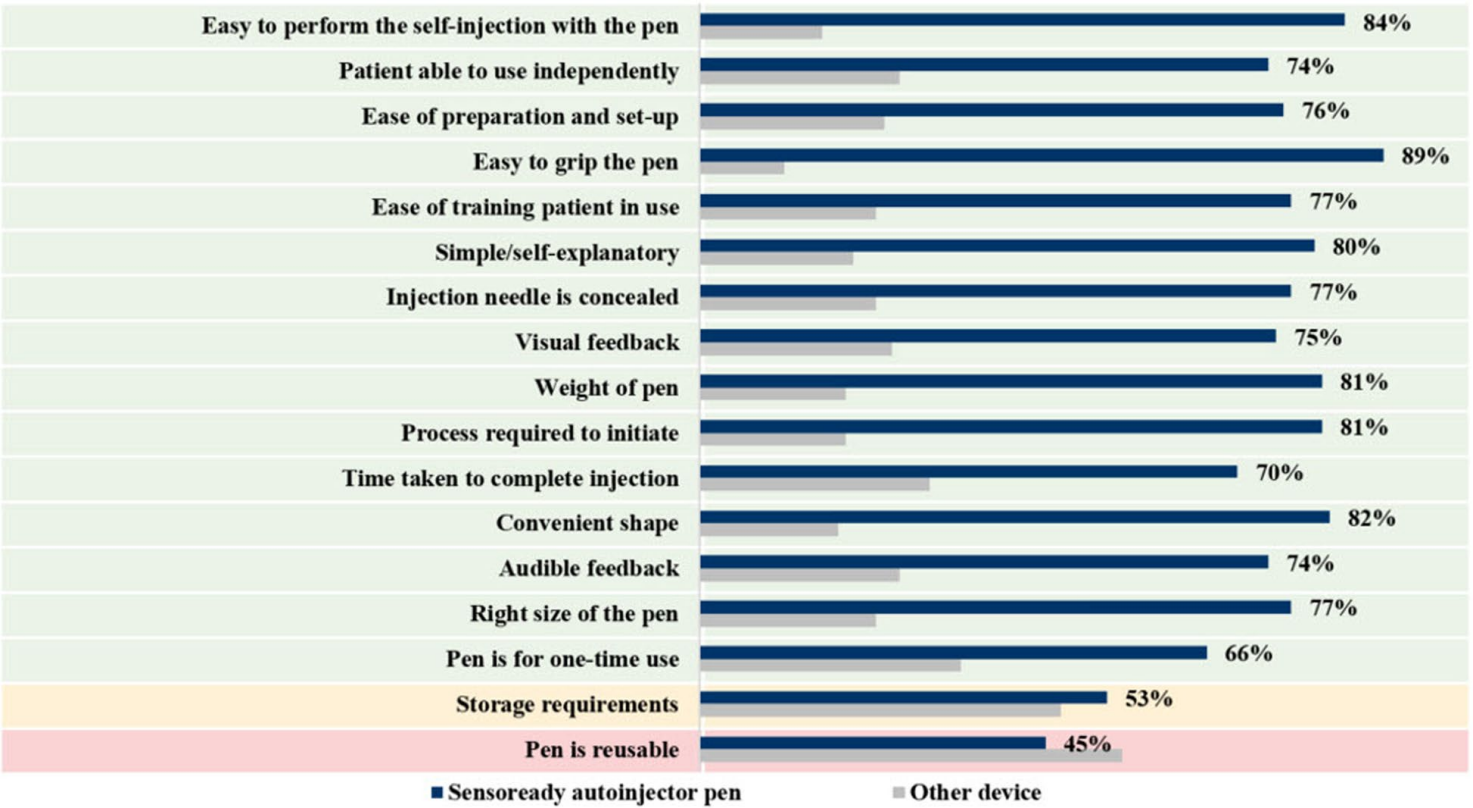
Attributes for which the Sensoready® autoinjector pen is preferred over other autoinjector devices. *Caution, Low base size. Ofatumumab, *n*=50; Rebif (Rebidose), *n*=16*; Avonex (Avonex Pen), *n*=27*; Copaxone (Autoject), *n*=34; Copaxone (YpsoMate), *n*=5*; Rebif (Rebismart), *n*=8*; Plegridy (Plegridy Pen), *n*=9*

For each of the prespecified attributes, a higher proportion of participants expressed a preference for the Sensoready® autoinjector pen over the comparator devices (Fig. 3). The preference rates for the Sensoready® autoinjector pen by attribute ranged from 45 to 89%, with the highest rates recorded for “easy to grip the pen” (89%), “easy to perform the self-injection with the pen” (84%), and “convenient shape” (82%). The highest preference rates recorded in the comparator device group were “pen is reusable” (55%) and storage requirement (47%).

**Fig. 4:**
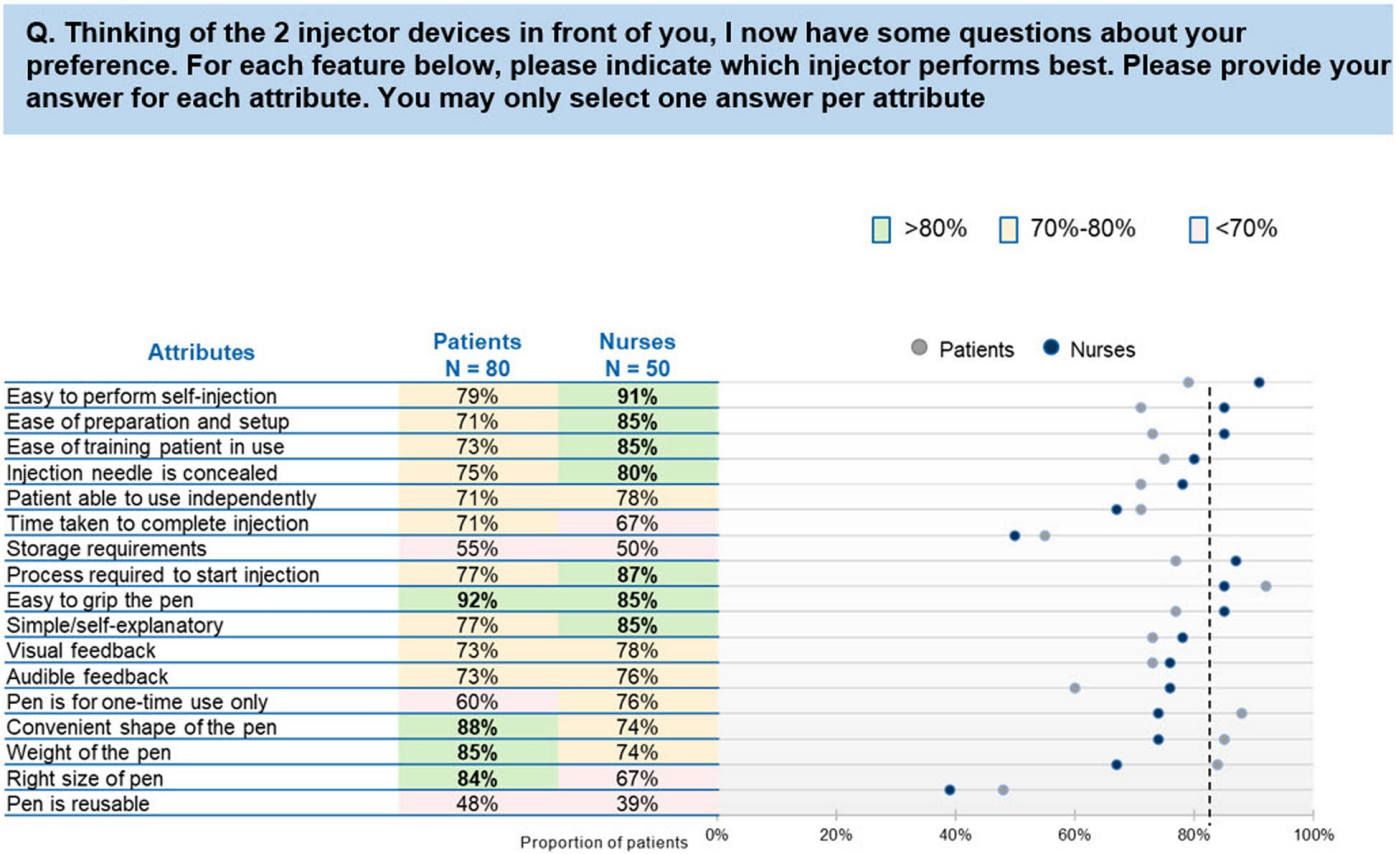
Patient and nurse ratings for the attributes for which Sensoready® autoinjector pen is preferred over other autoinjector devices. AI, autoinjector. Note: Other devices included were Rebif (Rebidose), Avonex (Avonex Pen), Copaxone (Autoject), Copaxone (YpsoMate), Rebif (RebiSmart), and Plegridy (Plegridy Pen). Of note, Sensoready® AI pen was not used to inject/self-administer medication during the survey nor did all the nurses and patients compare the Sensoready® AI pen against each of the other AI devices

Comparison of the nurse and patient ratings across the most important attributes showed that the preference rates for the Sensoready® autoinjector pen by nurses ranged from 39 to 91% (Fig. 4), with the highest rates recorded for “easy to perform self-injection with the pen” (91%) and “process required to start injection” (87%). The preference rates for the Sensoready® autoinjector pen by patients ranged from 48 to 92%, with the highest rates recorded for “easy to grip the pen” (92%) and “convenient shape of the pen” (88%).

### Overall device preference

**Fig. 5:**
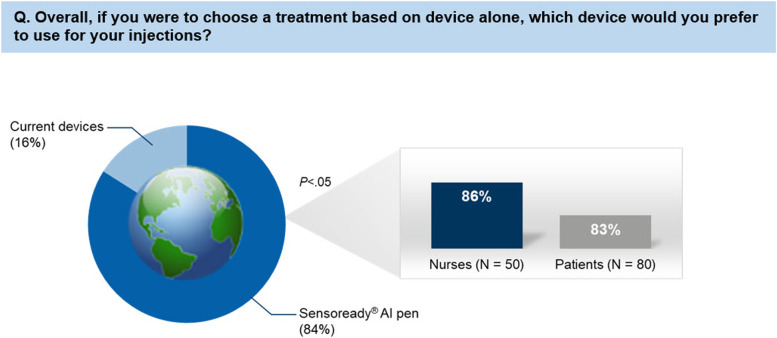
Preference of Sensoready® autoinjector compared with the other autoinjector devices. AI, autoinjector. Note: In this survey, questions comparing Sensoready® AI pen with other devices — namely Rebif (Rebidose), Avonex (Avonex Pen), Copaxone (Autoject), Copaxone (YpsoMate), Rebif (RebiSmart), and Plegridy (Plegridy Pen) — were included. Sensoready® AI pen was compared with ≥2 other devices by MS nurses (*N* = 50) and with their current device by MS patients (*N* = 80). Of note, Sensoready® AI pen was not used to inject/self-administer medication during the survey nor did all the nurses and patients compare the Sensoready® AI pen against each of the other AI devices

A majority of the participants preferred Sensoready® over their current device (84% vs. 16%; *p  *< 0.05), and the response was similar for both nurses (86%) and patients (83%; Fig. 5). Considering choice of treatment based on the device alone, 9/10 nurses and 8/10 patients preferred the Sensoready® autoinjector pen over their current device. More than 80% of patients and nurses rated the overall satisfaction with the Sensoready® autoinjector pen as “Very Good” or “Excellent”.

## DISCUSSION

Results of this multicenter survey suggest that both MS patients and nurses prefer Sensoready® autoinjector pen for subcutaneous administration of ofatumumab 20 mg over other autoinjectors for their current treatment(s), mostly driven by the ease of performing self-injection.

The survey reported patients able to use independently and easy to perform the self-injection with the pen were the most important attributes for both patients and nurses. For nurses, these attributes are crucial because the patients are able to inject independently and easily, which allows them to be in control of their treatment and have a positive impact on treatment adherence. For patients, they feel confident and comfortable administering the injection in their own homes or on the move without support from carers or nurses; moreover, the process of self-administration is easy and straight forward.

The results of the survey showed that the ofatumumab Sensoready® autoinjector pen achieved high scores across the most important attributes for both patients and nurses. The score was mainly driven by the preference around ease of use of the pen. Overall, 90% of patients and nurses believed that the Sensoready® autoinjector pen was easier to handle for patients than (their) current devices. Sensoready® device achieved significantly higher scores than Rebidose/Rebismart (Rebif), Avonex pen (Avonex), Autoject/YpsoMate (Copaxone), and Plegridy pen (Plegridy) across a variety of important attributes. Approximately 88% of nurses and 84% of patients believed that the Sensoready® autoinjector pen was the easiest to inject, mainly because of ease of use and the audible feedback obtained. Most of the participants (nurses, 87% and patients, 64%) preferred to start the injection by holding the device against the skin (i.e., buttonless injection start).

Perceptions regarding devices for MS available to date are mostly positive among both patients and nurses. For nurses, the ease of use of the Sensoready® device, the ability to be used independently by patients, ease of handling and operation because of substantial grip and light weight, and the novel shape facilitating holding of the device are thought to allow patients who may have mobility issues to hold the pen more easily than the traditional circle pens. Patients can be easily trained to use this device as it has a limited number of parts, and as the overall process is very straightforward, the patients are less likely to make mistakes. The acoustic and visual signals are both helpful for treatment administration and reassures completeness of treatment administration. The monthly frequency of injections is considered more favorable for patient compliance and relieving apprehension of injection; further, it is easier to store fewer pens.

For patients, the most important attributes were centered around maintaining their independence with their treatment with preparation and a device having minimal steps for set-up to ensure a smooth and error-free process. A majority of patients preferred to be able to hold the device against their skin versus the button as it gives them a higher level of control. The unconventional shape of the autoinjector pen, which is unique and triangular, makes it easy to handle by the majority of patients and is thought to aid the grip of the patient. Both audible and visual feedback are essential for patients and the Sensoready® pen positively differentiates the start and finish of the injection by the 2 clicks, and the visual indicator window provides a clear representation of the time taken to inject. In addition, a majority of the patients are attracted by the once-monthly dosage and consider it as a great advantage of the Sensoready® autoinjector pen. Patients felt that the set-up was simple, with limited number of parts, and very clear instructions. Although a small number of patients felt that the lack of a button was unconventional, it was not considered as a drawback because of the other positive features.

Features like shape, easy grip, buttonless injection, and ease of administration were more important for nurses than for patients. For nurses, the patients should be able to independently use an injectable device and have an easy training process, whereas for patients, a wider range of factors drove their preference. The patients’ preference centered around the ease of use of the device in terms of its shape and feedback mechanisms. These differences may reflect the difference in perspectives between nurses and patients. While assessing an injection device, nurses are more likely to refer to real-world experience in the clinic, whereas patients’ preference is centered around maintaining their independence with their treatment. These results may indicate that patients have a more concrete and sensorimotor understanding of “easy to perform the self-injection” than nurses.

The current survey results are consistent with the results of a previous study conducted in patients with rheumatoid arthritis indicated that the buttonless release of injection, better visual feedback because of a larger window, ease of grip, and a convenient triangular shape were some of the features of the Sensoready® autoinjector pen because of which it was preferred over other available autoinjector devices [21]. Patients using a buttonless autoinjector rated this attribute more highly than those using an autoinjector requiring to push a button, suggesting that preference for this feature is experience-dependent. Additionally, our results were also consistent with those reported in previous studies in that the ease of performing self-injection, a safely concealed needle preventing accidental injuries, and audible and visual feedback indicating that the dose has been completely injected were the most important features of an autoinjector [21, 26, 27].

Overall, the preference of the participants for Sensoready® autoinjector was ~ fivefold higher than that for the current device mainly because of the following advantages: (1) the short time required for administration of the injection (less than 5 min) was rated helpful by 65% nurses and 70% patients, (2) the frequency of monthly injection was a highly advantageous aspect of the Sensoready® autoinjector pen and was rated by over 90% of patients and nurses, and (3) not seeing the needle of the device had a greater impact on the nurses (84%) than on the patients (58%). The concealed needle provides protection for the patient, especially if they have a tremor. Furthermore, the hidden needle could be welcomed by naive patients, especially if they had needle phobia.

Further studies are required to explore the potential benefits of improved compliance using the autoinjector on disease outcomes. An ongoing study in the US should further evaluate the safety, tolerability, and usability of the autoinjectors in the real-world practice.

The drawbacks of the Sensoready® autoinjector pen include a concern expressed by some patients about the storage conditions required for the Sensoready® autoinjector pen as they may have limited fridge space. The ecological impact of a single-use pen is also a limitation for a number of patients and nurses globally.

Limitations of this pilot survey include a very small sample size. The research was conducted during the pandemic (for a small-time duration) which could have created a recruitment bias as we were unable to conduct the research in patients’ homes as originally planned. This survey was unable to compare the Sensoready® autoinjector pen in terms of overall convenience and ease of use of injectables with other routes of administration (e.g., orals and infusions), which is an important reality in MS. These different options may also have distinct ecological impacts. The usability parameters were tested independent of frequency which might impact on overall patient convenience over time. In addition, the analysis is all based on assumptions driven by the dummy demonstration device only which has no active medicine.

## Conclusions

In conclusion, the results of this survey showed that MS patients and nurses prefer the Sensoready® autoinjector pen for subcutaneous self-administration of ofatumumab over other autoinjectors for their current treatment. This positive perception regarding the Sensoready® autoinjector pen expressed by patients and nurses plays an important role in patient satisfaction and treatment adherence.

## Data Availability

The data that support the findings of this survey are available from [Novartis Pharma AG] but restrictions apply to the availability of these data, which were used under license for the current survey, and so are not publicly available. Data are however available from Dee Stoneman (dee.stoneman@novartis.com) upon reasonable request and with permission of [Novartis Pharma AG].

## Abbreviations

DMT: Disease-modifying therapy
MS: Multiple sclerosis
SPSS: Statistical Package For The Social Sciences
US: United States
